# Crotamine in *Crotalus durissus*: distribution according to subspecies and geographic origin, in captivity or nature

**DOI:** 10.1590/1678-9199-JVATITD-2019-0053

**Published:** 2020-04-06

**Authors:** Lídia J. Tasima, Caroline Serino-Silva, Daniela M. Hatakeyama, Erika S. Nishiduka, Alexandre K. Tashima, Sávio S. Sant’Anna, Kathleen F. Grego, Karen de Morais-Zani, Anita M. Tanaka-Azevedo

**Affiliations:** 1 Laboratory of Herpetology, Butantan Institute, São Paulo, SP, Brazil.; 2 Interinstitutional Postgraduate Program in Biotechnology (PPIB - IPT, IBU and USP), University of São Paulo(USP), São Paulo, SP, Brazil.; 3 Department of Biochemistry, Federal University of São Paulo (Unifesp), São Paulo, SP, Brazil.

**Keywords:** Rattlesnake, Snake venom, Toxins, Venom variation, Antivenom

## Abstract

**Background:**

*Crotalus durissus* is considered one of the most important species of venomous snakes in Brazil, due to the high mortality of its snakebites. The venom of *Crotalus durissus* contains four main toxins: crotoxin, convulxin, gyroxin and crotamine. Venoms can vary in their crotamine content, being crotamine-negative or -positive. This heterogeneity is of great importance for producing antivenom, due to their different mechanisms of action. The possibility that antivenom produced by Butantan Institute might have a different immunorecognition capacity between crotamine-negative and crotamine-positive *C. durissus* venoms instigated us to investigate the differences between these two venom groups.

**Methods:**

The presence of crotamine was analyzed by SDS-PAGE, western blotting and ELISA, whereas comparison between the two types of venoms was carried out through HPLC, mass spectrometry analysis as well as assessment of antivenom lethality and efficacy.

**Results:**

The results showed a variation in the presence of crotamine among the subspecies and the geographic origin of snakes from nature, but not in captive snakes. Regarding differences between crotamine-positive and -negative venoms, some exclusive proteins are found in each pool and the crotamine-negative pool presented more phospholipase A_2_ than crotamine-positive pool. This variation could affect the time to death, but the lethal and effective dose were not affected.

**Conclusion:**

These differences between venom pools indicate the importance of using both, crotamine-positive and crotamine-negative venoms, to produce the antivenom.

## Background

Pit vipers belonging to the genus *Crotalus*, popularly known as rattlesnakes, are present in all the Americas, whereas the Neotropical rattlesnake species (*Crotalus durissus*) can be found throughout most of South America [1]. This wide geographical distribution results in intraspecific variability of the venom composition, altering its effects and requiring different clinical treatments [2-6]. *C. durissus* is considered one of the most important species of venomous snakes in Brazil, due to the high mortality caused by its snakebites (1.1 %) [7]. 

**Figure 1.**
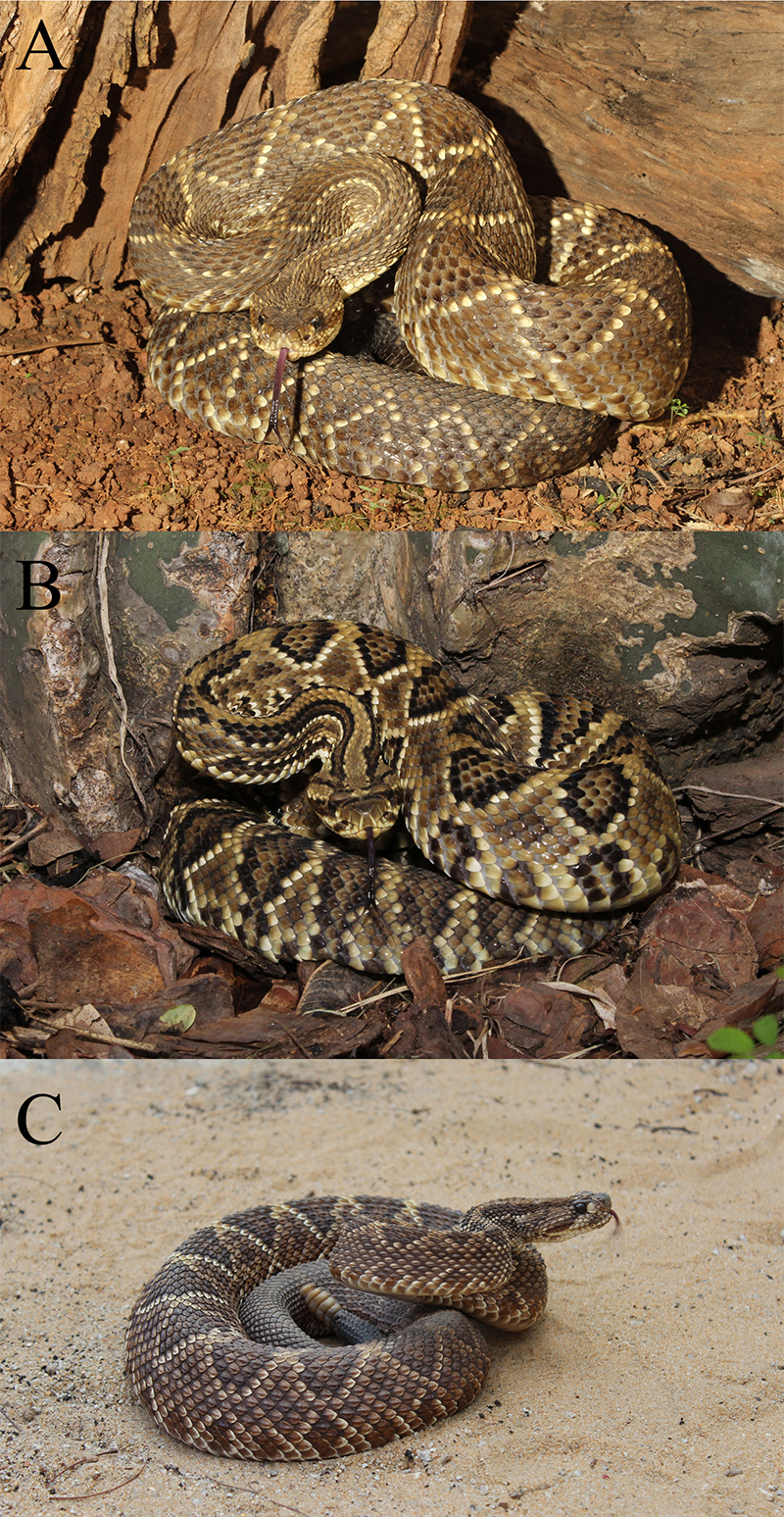
Subspecies of *Crotalus durissus* analyzed in the present study: **(A)** *C. d. terrificus*, **(B)** *C. d. collilineatus*, and **(C)** *C. d. cascavella*.

Even though it is the only species of this genus present in Brazil, different subspecies are recognized in accordance with geographical distribution. The taxonomy of this species has been revised and discussed by multiple authors [8]. Although the subspecies *C. d. terrificus*, *C. d. collilineatus* and *C. d. cascavella* are distinguished exclusively from each other by their morphology and geographical origin (Figure 1) [9], which is weakly substantiated, they are still considered subspecies by the Brazilian Herpetology Society [10]. Furthermore, these three subspecies are still kept apart in Butantan Institute for the crotalic antivenom production using immunized horses with a mixture composed of 50% *C. d. terrificus* and 50% *C. d. collilineatus* venoms.

The venom of *C. d. terrificus* contains four main toxins: crotoxin, convulxin, gyroxin and crotamine [11]. These components are responsible for the biological and toxic effects of the venom, whose main purpose is to weaken, paralyze, kill and digest prey [12,13]. Therefore, the venom can cause systemic neurotoxicity and myotoxicity, leading to progressive paralysis because of the high concentration of crotoxin [7]. Moreover, the myonecrotic toxin, crotamine, is also responsible for paralyzing the prey, avoiding both its escape and injuries to the snakes [14,15].

Crotamine was first observed in the venom of Argentinean rattlesnakes by Gonçalves and Polson [16] and, later on, was found in other rattlesnakes’ venom from southern Brazil [17]. Crotamine is a small basic myotoxin, with 4.8 kDa and isoelectric point around 10.8 [18]. It is constituted by a single chain of 42 amino acid residues [19] and contains three disulfide bonds [20]. The overall fold of crotamine is homologous to antimicrobial peptides belonging to the alpha-defensin, beta-defensin and insect defensin families [21-23]. This toxin induces myonecrosis [24], paralysis and extension of hind legs, and spontaneous and irregular contractions in the diaphragm of rats, mice and rabbits [25] by acting on sodium and potassium channels [26,27]. More recently, it has been demonstrated that crotamine possesses antitumoral [28], cytotoxic [29], antibacterial [30-32], anti-leishmanial [33], anthelmintic [34] and antimalarial effects [35]; as well as induces platelet aggregation [32].

Crotamine expression is not uniform within populations of snakes: it may have variable concentrations or even be absent in the venom of specimens of the same population. For example, concerning the Brazilian *Crotalus durissus* species, crotamine seems to be absent in *C. d. cascavella* [36-38]. In addition, Schenberg [39] observed a crotamine-positive distribution pattern in the states of São Paulo, Paraná and Minas Gerais. This observation is corroborated by Oguiura et al. [40], who found a relationship between a gene and the concentration of crotamine in snake venom, concluding that crotamine is a heritable character. This heterogeneity is of great importance in the view of antivenom production, because crotamine-positive and crotamine-negative venoms may have different mechanisms of action.

Both Calvete et al. [41] and Boldrini-França et al. [36] found a poor crotamine recognition by antivenom produced in Costa Rica and Butantan Institute. They suggest that the mixtures used on horse immunization to generate the serum did not contain enough amount of crotamine, resulting in ineffectiveness of antivenom to neutralize this protein during crotalic envenoming treatment. This suggestion instigated us to investigate the presence of crotamine in the venom of captive *C. durissus* of the Laboratory of Herpetology at Butantan Institute (CP), comparing them with the venom of newcomers from nature (NC), and to analyze the adequacy of CP venom for the pool composition to be used in antivenom production. We have also analyzed the correlation between the presence of crotamine in three subspecies (*C. durissus terrificus*, *C. durissus collilineatus* and *C. durissus cascavella*) (Figure 1) according to geographical distribution of the specimens. In addition, we compared the composition, lethality and neutralization of antivenom in crotamine-positive and crotamine-negative venom pools, searching for differences between these types of venoms.

## Methods

### Venoms and Antivenom

The venoms of *C. d. terrificus*, *C. d. collilineatus* and *C. d. cascavella* used in this study were obtained from the Laboratory of Herpetology at Butantan Institute (São Paulo, Brazil). Crude venoms of 110 captive (CP) and 115 newcomer (NC) adult snakes were collected by manual massage of the gland. Complete snake information is available in Additional file 1. Immediately after extraction, the samples were centrifuged at 2500 × g for 15 min, and the supernatant separated and kept at -20 ºC until testing. The crotalic antivenom (ACS - anticrotalic serum) was provided by the Butantan Institute (São Paulo, Brazil), produced by hyperimmunization of horses with a pool of two *Crotalus durissus* subspecies, namely *C. durissus terrificus* (50%) and *C. durissus collilineatus* (50%). About 10 mL of ACS neutralizes at least 15 mg of *Crotalus durissus terrificus* reference venom (serum neutralization in mice) according to the manufacturer.

### Animals

*In vivo* assays were performed in male Swiss mice (weight 20 ± 2 g) obtained from the Animal Breeding Center of Butantan Institute. The animal tests were approved by the Animal Care and Use Committee from Butantan Institute (n. 9485210217). All procedures involving animals were in accordance with the animal research ethical principles adopted by the Brazilian Society of Animal Science and the National Brazilian Legislation no 11.794/08.

### Identification of Crotamine-Positive and Crotamine-Negative Snake Venoms

*Polyacrylamide gel electrophoresis (SDS-PAGE)*

The amount of 20 μg of venom was analyzed by 15% SDS-PAGE, under reducing conditions, according to the method described by Laemmli [42]. Gels were fixed with 10% ethanol and 7% acetic acid for 1 hour and stained by Coomassie G according to manufacturer's recommendations (GE Healthcare). 

*Enzyme-linked immunosorbent assay (ELISA)*

The ELISA test was performed according to the method described by Oguiura et al. [18] with modifications. The venoms were diluted in 0.15 M NaCl for a concentration of 10 μg/mL. One hundred microliters of each sample were incubated for 2 h at 37°C in 96-well polystyrene plates. The plates were then blocked with 200 μL of phosphate-buffered saline (PBS - 137 mM NaCl, 2.7 mM KCl, 10 mM Na_2_HPO_4_, 2 mM KH_2_PO_4_, pH 7.4) containing 3% of bovine serum albumin (BSA) for 30 min at 37°C.

One hundred microliters of anti-crotamine antibody produced in rabbits (kindly provided by N. Oguiura [18]) (1/8,000 in incubation buffer - 10 mg/mL BSA in PBS) were added to each well. The plate was incubated for 1 hour at 37°C. Another 100 µL of secondary antibody (anti-rabbit IgG-peroxidase conjugate 1/3,000 in incubation buffer) was added to each well and the plate was incubated at 37°C for 1 hour. Between each step, the plate was washed three times with wash buffer (PBS containing 0.1% Tween 20).

The volume of 100 μL of developing solution (OPD 1 mg/mL in PBS containing 0.1% H_2_O_2_) was added per well and the plate was incubated for 20 min at room temperature and protected from light. The reaction was stopped with 50 μL of 30% H_2_SO_4_. The absorbance was recorded at 492 nm in microplate reader i3 SpectraMax (Molecular Devices). A crotamine curve ranging from 0.016 to 1 μg/mL was used to quantify the amount of crotamine present in each sample.

*Western blotting*

Venom samples (20 µg) separated by SDS-PAGE were electrotransferred using Trans-Blot Turbo Transfer System (Bio-Rad) onto PVDF membranes, previously equilibrated in transfer buffer (25 mM Tris, 192 mM glycine, 20% ethanol). It was used constant current of 2.5 A and voltage up to 25 V for 5 min. Thereafter, the membrane was blocked with TBS-milk (Tris-buffered 0.15 M NaCl containing 5% non-fat milk and 0.1% Tween 20) overnight at 4°C. The membrane was washed twice with wash buffer (10 mM Tris, 150 mM NaCl, 0.1% Tween 20; pH = 7.5) and incubated with 1/2,000 first antibody (anti-crotamine) for 2 h at 4°C. After three times washing using wash buffer, the membrane was exposed to 1/10,000 anti-rabbit conjugated to peroxidase (Sigma) for 2 h at 4°C. The membrane was washed three times again as abovementioned and reaction was developed with diaminobenzidine (Sigma) and H_2_O_2_ [43].

### Localization by DIVA-GIS Program

Information regarding the presence/absence of crotamine in individual venom samples was charted according to the geographic localization of the respective animals, using DIVA-GIS program.

### Comparison between crotamine-positive and crotamine-negative venoms

After defining the crotamine-positive and crotamine-negative snake venoms, we made a pool of crotamine-positive venoms and another of crotamine-negative venoms, containing 10 µL of each snake venom. Therewith, each pool had the same proportion of CP and NC snake venoms selected randomly regardless of gender or geographic distribution, totalizing 24 venoms of different snakes in each pool. The pools were lyophilized and stored at -20ºC until use. The selected snakes are bolded on Additional file 1.

*Reversed-phase high-performance liquid chromatography (RP-HPLC)*

About 3 mg of each freeze-dried venom pool were dissolved in 400 µL of solution A (0.1% trichloroacetic acid - TFA) and centrifuged at 10,000 rpm for 10 minutes. Following a method previously described by Calvete et al. [44], the supernatant was applied on a Teknokroma Europa 300 C-18 (25 x 0.46 mm, 5 μm particle size, 300 Å pore size) reverse-phase column, previously equilibrate with solution A + 5% solution B, using the ÄKTA Purifier UPC10 system (GE Healthcare). Samples were eluted in solution B (0.1% TFA and 95% acetonitrile) under the following conditions: 5% of solution B for 5 min, a gradient of 5-25% of solution B for 10 min, a gradient of 25-45% of solution B for 60 min, a gradient of 45-70% of solution B for 10 min, a gradient of 70-100% of solution B for 10 min, and more 10 min with 100% of solution B. Fractionation was carried out at flow rate of 1 mL/min and the elution of protein peaks was monitored at 280 nm and 215 nm.

*In solution trypsin digestion and mass spectrometric identification*

Samples of 100 µg of pooled venoms were dissolved in 50 mM NaHCO_3_, reduced with 5 mM dithiothreitol for 25 min at 60°C and then alkylated in the dark with 14 mM iodoacetamide at room temperature for 30 min. Proteins were digested using trypsin (Sigma-Aldrich) at a 1:100 (w/w) enzyme:substrate ratio, overnight at 37°C. Digestion was stopped by addition of 0.6% TFA (final concentration), samples were dried using a vacuum centrifuge and stored at -20ºC until use [45]. 

Mass spectrometry experiments of venom digests were performed on a Synapt G2 HDMS (Waters) mass spectrometer coupled to a nanoAcquity UPLC system (Waters). Approximately 5 µg of each peptide mixture was loaded online for 5 min at a flow rate of 8 µL/min of phase A (0.1% formic acid) using a Symmetry C18 trapping column (5 µm particles, 180 µm x 20 mm length; Waters). The mixture of trapped peptides was subsequently separated by elution with a gradient of 7-35% of phase B (0.1% formic acid in acetonitrile) through a BEH 130 C18 column (1.7 µm particles, 75 x 150 mm; Waters) in 90 min, at 275 nL/min. Data were acquired in the data-independent mode UDMSE [46] with ion mobility separation in the m/z range of 50-2000, in the resolution mode and with 1.25 s of scan time. The ESI source was operated in the positive mode with a capillary voltage of 3.1 kV, block temperature of 100°C, and cone voltage of 40 V. 

For lock mass correction, a [Glu1]-Fibrinopeptide B solution (500 fmol/mL in 50% acetonitrile, 0.1 formic acid; Peptide 2.0) was infused through the reference sprayer at 500 nL/min and sampled every 60 s. Venom samples were analyzed in technical duplicates. Mass spectrometry raw data were processed in ProteinLynx Global Server 3.0.3 (PLGS, Waters) using low energy threshold of 750 counts and elevated energy threshold of 50 counts. 

Database searches were performed against *Crotalus durissus* sequences from UniprotKB/Swissprot (www.uniprot.org, 295 sequences, downloaded in May 22, 2019). The following search parameters were used: automatic tolerances for precursor and fragment ions, carbamidomethylation of cysteine as fixed modification and oxidation of methionine, N-terminal acetylation, glutamine and asparagine deamidation as variable modifications. Up to two missed cleavage sites were allowed for trypsin digestion. Protein identifications were considered with a minimum of one fragment ion per peptide, five fragment ions per protein, two peptides per protein and a false discovery identification rate set to 1%, estimated by a simultaneous search against a reversed database [47]. 

Label-free quantification was performed in Progenesis QI for Proteomics (NonLinear Dynamics, Newcastle, UK) as previously reported [48]. Briefly, the raw files were loaded in the software and a reference run for the replicates was automatically chosen. Precursor ion retention times were processed for alignment, peak picking and normalized to the reference run with default parameters. Relative quantification was carried out by the comparison of peptide ion abundances, which were calculated as the sum of the areas under the isotope boundaries. Significance of the differentially abundant proteins between the groups was determined using unpaired Student’s t-test considering p < 0.05.

*Lethal dose 50% (LD*_*50*_ *)*

Venom lethality was evaluated by injecting different doses (0.5 μg-5.0 μg; dilution factor: 1.8) of the crotamine-positive or crotamine-negative pools dissolved in 500 μL of 0.15 M NaCl by the intraperitoneal route, in groups of male Swiss mice (n = 5 of each dose). Deaths were recorded during 48 h and the LD_50_ was calculated using the Probit analysis method [49].

*Effective dose 50% (ED*_*50*_ *)*

The ED_50_ is defined as the dose that is able to neutralize the action of venom in 50% of the same population of mice, based on the amount of venom of 3-5 LD_50_.

The venom (4x LD_50_) and serial dilutions of anticrotalic antivenom (Butantan Institute 15052) were homogenized and incubated at 37°C for 30 minutes. Following, Swiss mice groups (n = 5) were inoculated intraperitoneally (500 μL per mouse). The first group was injected with enough serum to fully neutralize the amount of injected venom. Deaths were recorded during 48 h and the ED_50_ was calculated using the Probit analysis method [49]. The ED_50_ was expressed as μL antivenom/μg venom.

### Statistical analyses

We used Chi-square test to compare the methodologies and the presence of crotamine between CP and NC, among subspecies. Student's t-test was employed to compare the LD_50_, ED_50_ and quantitative proteomics. Analyses were performed using GraphPad Prism 7 program. Differences with p < 0.05 were considered statistically significant.

## Results

### Identifying crotamine-positive and crotamine-negative snake venoms

**Figure 2.**
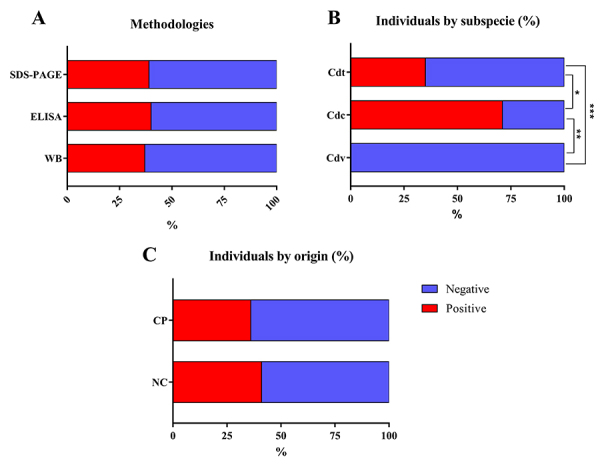
Percentage of *C. durissus* crotamine-positive (red) and crotamine-negative (blue) identified by: **(A)** each methodology, **(B)** according to subspecies and **(C)** to the origin of snakes. WB: western blotting; Cdt: *C. d. terrificus*; Cdc: *C. d. collilineatus*; Cdv: *C. d. cascavella*; CP: captivity snakes; NC: newcomer snakes. *p < 0.0003; **p < 0.0001; ***p < 0.02.

Three different methods were used in the present work - namely SDS-PAGE, ELISA and western blotting - to verify the presence of crotamine in *C. durissus* snakes and correlate the presence of this protein within subspecies and geographic distribution. Regarding methodology, no significant difference was observed among the results obtained by each one (Figure 2A). For comparison purposes, samples that showed divergent results among the three approaches were excluded from other comparisons.

According to subspecies, most crotamine-positive snakes (71%) were *C. d. collilineatus*, whereas all *C. d. cascavella* were crotamine-negative. Only 35% of *C. d. terrificus* individuals were crotamine-positive (Figure 2B). No statistical difference was found when crotamine-positive snakes were compared between NC and CP groups.

**Figure 3.**
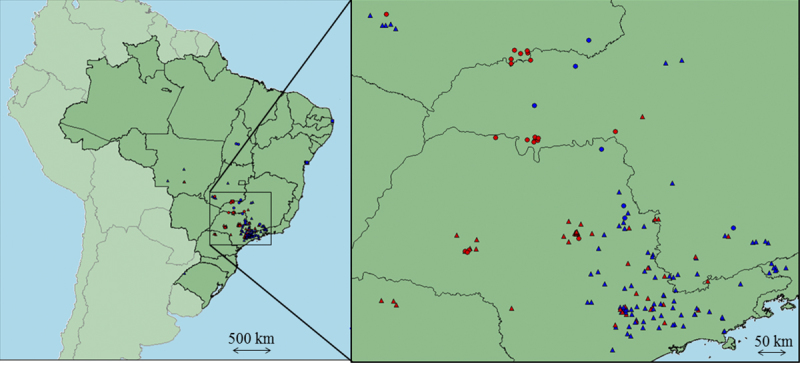
Geographic distribution of *C. durissus* crotamine-positive (red) and crotamine-negative (blue) in a region of southeast of Brazil. ▲: *C. d. terrificus*; ●: *C. d. collilineatus*.

The geographic distribution of snakes in accordance with presence of crotamine showed a higher concentration of crotamine-positive snakes in the northwest region of São Paulo state whereas crotamine-negative animals are found predominantly in the northeast region (Figure 3).

The final result for each sample, and images of SDS-PAGE and western blotting membranes can be seen in Additional files 1, 2 and 3, respectively.

### Differences between crotamine-positive and crotamine-negative venoms

*Polyacrylamide gel electrophoresis (SDS-PAGE)*

**Figure 4.**
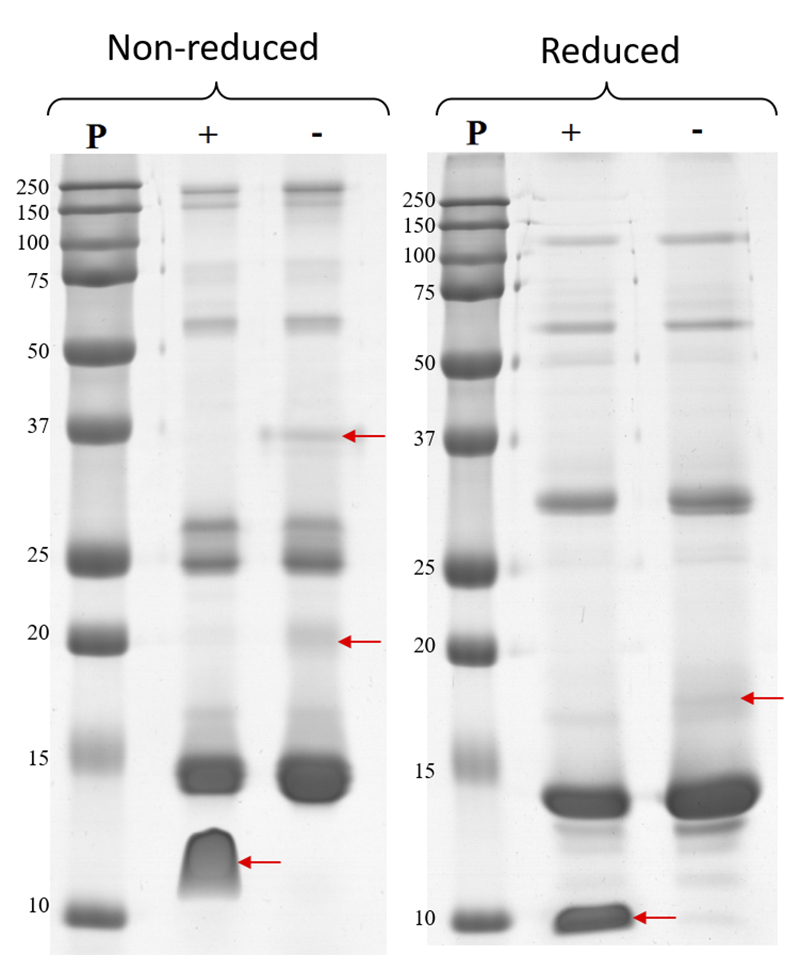
Electrophoretic profile of crotamine-positive pool (+) and crotamine-negative pool (-) from *C. durissus* venom under reduced (left panel) and non-reduced (right panel) conditions. Venom pools (20 µg) were subjected to 15% SDS-PAGE and proteins were stained using Coomassie G (GE Healthcare). Arrows point to different bands between crotamine-positive and crotamine-negative venom pools. MW: molecular weight marker (Dual Color Precision Plus Protein Standards - BioRad).

SDS-PAGE results showed few differences between crotamine-positive and crotamine-negative pools (Figure 4). Crotamine-negative pool under non-reducing conditions showed two exclusive bands (approximately 20 kDa and 37 kDa), and only one under reducing condition (17 kDa). Arrows in Figure 4 indicate differential protein bands.

*Reversed-phase high-performance liquid chromatography (RP-HPLC)*

**Figure 5.**
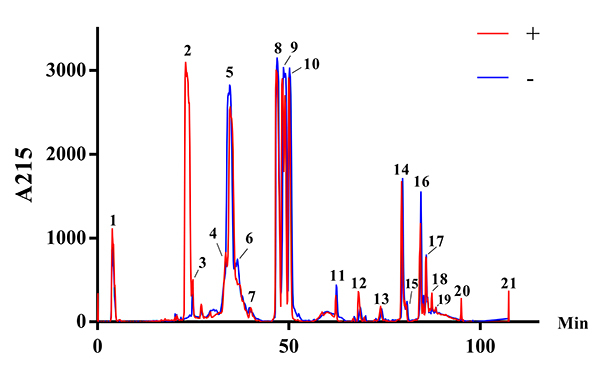
Elution profiles of pools of *C. durissus* venom by RP-HPLC monitored by 215 nm. Samples of 3 mg of lyophilized venom pools were dissolved in 0.1% trifluoroacetic acid (TFA) (solution A) and subjected to RP-HPLC on a C18 column. Elution was performed at 1.0 mL/min by applying a gradient toward 0.1% TFA and 95% acetonitrile (solution B), as described in the experimental section. Red (+): crotamine-positive venom pool; blue (-): crotamine-negative venom pool.

**Figure 6.**
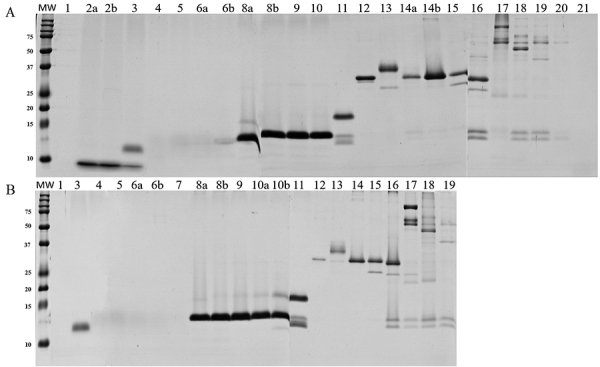
Polyacrylamide gel electrophoresis (15%) of peaks collected by HPLC. The numbers correspond to numbered chromatographic peaks shown in Figure 7. Peaks divided into two aliquots are represented by letters “a” and “b”. MW: molecular weight marker (Dual Color Precision Plus Protein Standards - BioRad); upper panel: crotamine-positive venom pool (+); bottom panel: crotamine-negative venom pool (-).

Venoms were fractionated by RP-HPLC (Figure 5), and each peak was then separated by SDS-PAGE (Figure 6). The chromatograms were similar to each other, except for the peak related to crotamine, the one with the most striking difference (peak 2 in Figure 5).

*Mass spectrometric identification (MS)*

**Figure 7.**
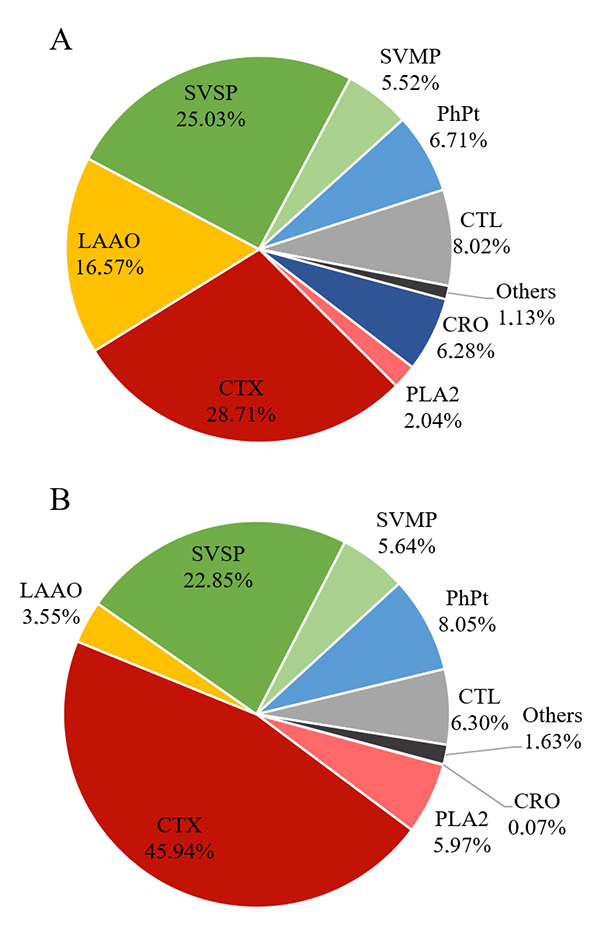
Overall composition estimated by mass spectrometry of venom pools from *C. durissus* according to protein families, expressed as percentages. **(A)** Crotamine-positive venom pool, **(B)** crotamine-negative venom pool. Protein family abbreviation - CRO: crotamine; PLA_2_: phospholipase A_2_; CTX: crotoxin; LAAO: L-amino acid oxidase; SVSP: snake venom serine protease; SVMP: snake venom metalloproteinase; PhPt: phosphoprotein; CTL: C-type lectin. Others - globin: fragment of globin HBD, BPP: bradykinin potentiating peptide, PLI: phospholipase A_2_ inhibitor; VNGF: venom nerve growth factor.

The MS showed a difference between pools in relation to the abundance of some protein families (Figure 7), but none of the pools showed any exclusive protein ( Additional file 4). The most relevant differences were in the higher abundance of CTX and PLA_2_ of crotamine-negative pool, with 45.94% and 5.97%, respectively, while the positive-pool presented 28.71% of CTX and 2.04% of PLA_2_. Moreover, the crotamine-positive pool had a higher abundance of LAAO (16.57%) than the crotamine-negative one (3.55%).

*Lethal dose 50% (LD*_*50*_ *))*

The calculated LD_50_ of pools were resembling. The LD_50_ of crotamine-negative pool was 1.59 μg/20 g animal (confidence interval: 1.19-2.13 μg/20 g animal), and the crotamine-positive pool dose was 1.75 μg/20 g animal (confidence interval: 1.26-2.44 μg/20 g animal).

*Effective dose 50% (ED*_*50*_ *)*

The ED_50_ of pools were very similar to each other. The ED_50_ of crotamine-positive pool was 0.29 µL/μg venom (confidence interval: 0.27-0.31 µL/μg venom) and the ED_50_ of crotamine-negative pool was 0.38µL/μg venom (confidence interval 0.28-0.59 µL/μg venom).

## Discussion

### Identifying crotamine-positive and crotamine-negative snake venoms

In the present work, the authors used three different methods to verify the presence of crotamine in *C. durissus* snakes of different origins and subspecies. Our data showed that there is no significant difference among the results obtained by each methodology (Figure 2A). However, both western blotting and ELISA assay require the production of specific antibodies in laboratory animals, since they are not commercially available [50]. Thereby, a *C. durissus* snake selection system based on the detection of crotamine in its venom by SDS-PAGE could be implemented in the Laboratory of Herpetology at Butantan Institute to include in the serpentarium a determined percentage of crotamine-positive snakes, assuring the presence of this protein in the mixture used in antivenom production.

For comparison purposes, only samples identified as crotamine-positive or crotamine-negative by all three methodologies (212 venom samples) were used for comparative analysis regarding subspecies and geographic distribution of the specimens. Among these venom samples, 131 were crotamine-negative, corresponding to 62% of the total samples analyzed. A comparable result was obtained by Lourenço et al. [51], but this majority of crotamine-negative snakes could be due to the higher number of samples of some specific geographic region or subspecies.

### Comparison among subspecies

When considering subspecies, all *C. d. cascavella* used in this work were crotamine-negative (Figure 2B), corroborating the results obtained by Boldrini-França et al. [36]. In addition, Toyama et al. [37] analyzed venoms of *C. d. cascavella* from three different regions of Brazilian northeast and only found crotamine in the venom of snakes belonging to Fortaleza city. This could explain the absence of crotamine in our samples, since the specimens used in this work were not from Fortaleza.

*C. d. collilineatus* was the subspecies with the highest percentage of crotamine-positive individuals, with 71% crotamine-positive snakes (Figure 2B). It is worth mentioning that 94% of *C. d. collilineatus* used in this study are from captivity. Only two snakes were NC and from these, only one was crotamine-positive. Oliveira et al. [52] found a different result: only one of 22 venoms of *C. d. collilineatus* studied showed crotamine in its composition.

The subspecies *C. d. terrificus* had the largest number of individuals; this is the subspecies from nature more often donated to the Butantan Institute, totalizing 66 captive and 104 wild snakes, from which 35% of individuals are crotamine-positive. Considering only CP snakes, only 26% have crotamine in its venom. This result shows that the amount of crotamine-positive *C. d. terrificus* is low and may contribute for the discrepancy of this protein in the pool used for anticrotalic antivenom production.

Santoro et al. [38] had already compared the venoms of these three subspecies and concluded that the venoms are very similar, although *C. d. cascavella* venom was the most different one. Corroborating the findings of the present work, these authors did not find crotamine in *C. d. cascavella* venom. This result was also found by Rangel-Santos et al. [53], whose work did not identify considerable differences among activities of these three subspecies of *C. durissus*.

### Comparison between CP and NC

Previous studies indicate that captivity may alter the composition or activities of snake venoms, as shown by Freitas-de-Sousa et al. [54], who compared venoms of captive and wild *Bothrops atrox* snakes and observed some differences between these two groups. On the other hand, McCleary et al. [55] have not identified significant differences between captive and wild *Pseudonaja textilis* snake venoms. In addition, our group has recently shown that captivity does not influence the composition and activities of *B. jararaca* snake venom [56,57]. 

In the present work, a higher percentage of crotamine-positive snakes was found in NC (41%) in comparison to CP (36%) (Figure 2C). However, no statistical difference was found when these data were analyzed by Chi-square test. Even though these values are comparable, the low percentage of CP crotamine-positive snakes may contribute for the weak immunorecognition capability of the anticrotalic antivenom toward crotamine [58]. Another possible cause of anticrotalic antivenom deficiency regarding crotamine immune recognition is the low immunogenicity of crotamine, due to its low molecular weight [59-62]. However, Boldrini-França et al. [36] showed that crotamine is able to induce a strong immune response after immunizing rabbits against crotamine-positive snake venom.

### Geographic distribution

The geographic distribution of crotamine-positive and crotamine-negative snakes revealed a pattern (Figure 3). Most of the snakes collected are from the state of São Paulo, so the standardized distribution in this state is more visible. There is a higher concentration of crotamine-positive snakes in the northwest region of São Paulo whereas crotamine-negative are found predominantly in the northeast region. This result is corroborated by Schenberg [39], who found a similar distribution pattern of crotamine in *C. durissus* snakes in the same region. 

### Differences between crotamine-positive and crotamine-negative venoms

SDS-PAGE results showed other differences in addition to the crotamine band (between 15 kDa and 10 kDa under non-reducing conditions and above 10 kDa under reducing conditions) in the electrophoretic profile of crotamine-negative and crotamine-positive pools (Figure 4). Crotamine-negative pool under non-reducing conditions shows two bands not found in the crotamine-positive pool: one with approximately 20 kDa and another close to 37 kDa. Under reducing conditions, the crotamine-negative pool shows one band with 17 kDa not found in the crotamine-positive venom pool, which is probably the same protein that appears in the non-reduced state. Despite these differences, no exclusive protein was found in either pool ( Additional file 4). It is important to consider that these proteins may only be present in some crotamine-negative individuals but not others, not necessarily representing a general role. 

Much like SDS-PAGE, the overlapping HPLC chromatograms showed higher similarity between the pools (Figures 5 and 6), with the crotamine peak being the most notable difference (peak 2, Figure 5).

The relative abundance of proteins that compose each venom pool was estimated by shotgun mass spectrometric analysis (MS) (Figure 7). *Crotalus durissus* snake venom is composed mainly by crotoxin (CTX), a heterodimeric phospholipase A_2_ (PLA_2_) and the main responsible for the neurotoxic effect of the venom [41]. In addition to crotoxin, other PLA_2_s are present in the *C. durissus* venom composition. Besides neurotoxicity, PLA_2_s in general also cause cardiotoxicity, myotoxicity, hemorrhage, as well as edema, convulsions, hyperalgesia, inﬂammation, hypotension, inhibition of platelet aggregation, anticoagulation and hemolysis [63]. In the crotamine-positive pool, the relative abundance of CTX and PLA_2_ is lower than in the crotamine-negative pool, with 28.71% and 45.94% of CTX, and 2.04% and 5.97% of PLA_2_, respectively.

Identified as the second most abundant component of *C. durissus* venom, snake venom serine proteases (SVSP) affect the victim hemostasis, disturbing mainly the coagulation system [63]. The crotamine-positive venom pool has slightly higher abundance of SVSP than crotamine-negative pool (25.03% and 22.85%, respectively).

L-amino acid oxidase (LAAO) amount was strikingly different between the pools, with crotamine-positive pool containing almost five times more LAAO than crotamine-negative pool. Although its role in envenoming is still not clear, interesting biological activities have been related for this protein, known to be responsible for the yellow color of the venom [64-66]. This difference could be caused by the venoms selected to compose the crotamine-positive pool that included more yellow venoms than the ones selected for the crotamine-negative pool ( Additional file 1). In addition, crotamine-positive pool showed a higher amount of C-type lectin (CTL). 

Phosphoprotein (PhPt) found in both venoms is originated by ophidian paramyxovirus, that can be found in sputum of infected *C. durissus* [67]. Probably, these viruses were present in the saliva of wild snakes and the virus was collected with the venom. Snake venom metalloproteases (SVMP) are in comparable relative abundances in both pools and were found in low quantities, corroborating the findings of other authors [41]. The category “others” includes fragment of globin HBD, bradykinin potentiating peptides (BPP), phospholipase A_2_ inhibitor (PLI) and venom nerve growth factor (VNGF). No exclusive protein was found in none of the pools. The complete MS results are found in Additional file 4.

**Figure 8.**
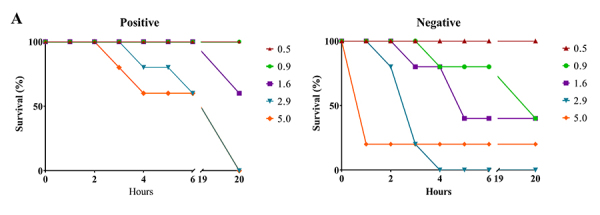
Lethal dose of venom pools from *C. durissus*. Survival of mice of different groups according to time to death after injection of **(A)** crotamine-positive and **(B)** crotamine-negative venom pools. Different colors represent different doses (µg venom/animal).

As for LD_50_, crotamine-negative venom pool was similar to crotamine-positive pool. The LD_50_ found by Santoro et al. [38] for *C. d. terrificus* (1.468 μg/20 g animal) was closer to the LD_50_ of the crotamine-negative pool (1.59 μg/20 g animal), whereas the crotamine-positive pool dose was slightly higher (1.75 μg/20 g animal). The similarity of LD_50_, despite the differences in composition, showed that synergism between proteins is more important for lethality than individual protein activities. Notwithstanding, the crotamine-positive venom pool took longer to kill than the crotamine-negative pool (Figure 8A and B). 

The animals were observed for a total period of 48 hours after venom injection and the number of dead animals per group was recorded every hour until 6 hours and then every 24 hours after venom injection. After the first 24 hours post-injection, there were no further deaths in both groups. In the first two hours, five animals injected with the crotamine-negative pool died, while all mice injected with the crotamine-positive pool were still alive. Most deaths caused by the crotamine-positive venom pool were recorded 24 hours after injection, while most animals injected with the crotamine-negative venom pool died within the first 6 hours. This difference in time to death may be due to the presence of crotamine, which is a slightly lethal protein [58,68,69]. Moreover, crotamine-negative venom pool possesses relatively more CTX and PLA_2_, the clinically most important neurotoxins of *C. durissus* [70], than crotamine-positive venom pool (Figure 7) [36,41]. This observation suggests that envenoming caused by crotamine-negative *C. durissus* snakes may develop a more severe and faster symptomatology than those caused by crotamine-positive specimens. 

**Figure 9.**
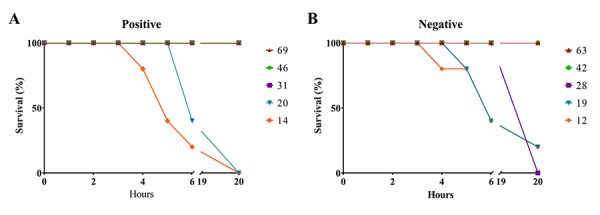
Effective dose of venom pools from *C. durissus*. Survival of mice of different groups according to time to death after injection of **(A)** crotamine-positive and **(B)** crotamine-negative venom pools pre-incubated with crotalic antivenom. Different colors represent different doses (µL antivenom/animal).

As well as the lethal dose, ED_50_ showed no statistically significant difference between pools. The ED_50_ of crotamine-positive pool (0.29 µL/μg venom) is somewhat lower than the crotamine-negative pool (0.38µL/μg venom). ED_50_ of both pools found in the present study comprised a better result when compared to that of the literature (0.5 µL antivenom/μg venom) [4]. Time to death did not show a wide difference between pools (Figure 9A and B). Despite the poor recognition of crotamine by antivenom [36,41], the serum did not demonstrate efficiency differences to neutralize the mortality of both pools, probably due to the low relevance of this protein to venom lethality.

Finally, combined, these results could emphasize that, although the presence of crotamine is important to compose the venom pool used to produce crotalic antivenom, our findings suggest that crotamine-negative venom causes death in mice faster than crotamine-positive venom. Thus, it would be important to carefully determine the snake composition of the serpentarium of venom producer facilities, considering not only the crotamine, but also all the complex set of proteins that compose the venom.

## Conclusion

There are several studies on the variation of the biological activity and the composition of snake venoms and their importance to antivenom production. In the present work, we analyzed the crotamine variation of *C. durissus* venoms and sought to relate this variant to the subspecies origin (captivity or nature) and geographic localization. A possible correlation was found between the presence of crotamine in relation to subspecies, in which *C. d. collilineatus* is predominantly crotamine-positive, *C. d. cascavella* does not present crotamine and *C. d. terrificus* is mostly crotamine-negative. There is also a possible population correlation, in which populations of *C. durissus* of the northwest of São Paulo possess more snakes with crotamine than those found in the southeast. Moreover, there is no correlation between snakes from captivity or from the wild and the presence of crotamine in their venoms. 

Despite the determination to use only crotamine-positive snakes for antivenom production by the Brazilian Ministry of Health [71], our findings show differences between pools in abundance of some proteins in venom composition and time to death in the lethal dose assay. This indicates the importance to use both crotamine-positive and crotamine-negative venoms to produce the antivenom.

### Abbreviations

ACS: anticrotalic serum; BPP: bradykinin potentiating peptide; BSA: bovine serum albumin; CP: captive snakes; CRO: crotamine; CTL: C-type lectin; CTX: crotoxin; ED_50_: effective dose 50%; ELISA: enzyme-linked immunosorbent assay; LAAO: L-amino acid oxidase; LD_50_: lethal dose 50%; MS: mass spectrometric analysis; NC: newcomers snakes; OPD: o-phenylenediamine dihydrochloride; PBS: phosphate-buffer saline; PhPt: phosphoprotein; PLA_2_: phospholipase A_2_; PLGS: ProteinLynx global server; PLI: phospholipase A_2_ inhibitor; RP-HPLC: reversed-phase high-performance liquid chromatography; SDS-PAGE: polyacrylamide gel electrophoresis; SVMP: snake venom metalloproteases; SVSP: snake venom serine protease; TBS: tris-buffer saline; TFA: trichloroacetic acid; VNGF: venom nerve growth factor.

## Supplementary material

The following online material is available for this article:

Additional file 1. Information regarding subspecies, gender, geographic origin, venom color and the presence of crotamine in venom of *C. durissus* used throughout this study.

Additional file 2. Polyacrylamide gel electrophoresis (15%) of all *C. durissus* used in this work. The numbers of samples correspond to snakes informed in Additional file 1.

Additional file 3. Western blotting of all *C. durissus* used in this work. The numbers of samples correspond to snakes informed in Additional file 1.

Additional file 4. Proteins identified in crotamine-positive and crotamine-negative venom pools of *C. durissus*. Quantitative data were obtained through label-free quantification by high resolution LC-MS/MS considering the averaged intensities of the three most intense peptides of each identified protein. Differentially abundant proteins with statistical significance (fold change ≥ 1.5 or ≤ 0.67; p < 0.05) are bolded.

## References

[B1] Campbell JA, Lamar WW (1989). The venomous reptiles of Latin America.

[B2] Chippaux JP, Williams V, White J (1991). Snake venom variability: methods of study, results and
interpretation. Toxicon.

[B3] Francischetti IMB, Gombarovits MEC, Valenzuela JG, Carlini R, Guimara JA (2000). Intraspecific variation in the venoms of the South American
rattlesnake (Crotalus durissus terrificus). Comp Biochem Physiol C Toxicol Pharmacol.

[B4] Saravia P, Rojas E, Arce V, Guevara C, López JC, Chaves E (2002). Geographic and ontogenic variability in the venom of the
neotropical rattlesnake Crotalus durissus: pathophysiological and
therapeutic implications. Rev Biol Trop.

[B5] Dos-Santos MC, Assis EB, Moreira TD, Pinheiro J, Fortes-Dias CL (2005). Individual venom variability in Crotalus durissus ruruima snakes,
a subspecies of Crotalus durissus from the Amazonian region. Toxicon.

[B6] Borja M, Neri-Castro E, Castañeda-Gaytán G, Strickland J, Parkinson C, Castañeda-Gaytán J (2018). Biological and proteolytic variation in the venom of Crotalus
scutulatus scutulatus from Mexico. Toxins (Basel).

[B7] Cardoso JLC, de S. França FO, Fan HW, Málaque CMS, Haddad V (2009). Animais Peçonhentos no Brasil. Biologia, Clínica e Terapêutica dos
Acidentes.

[B8] Uetz P., Freed P., Hošek J (2019). The Reptile Database.

[B9] Hoge AR, Romano-Hoge S (1978). Synopsis of the Poisonous Snakes from Brazil. Mem Inst Butantan.

[B10] Costa HC, Bérnils RS (2018). Répteis do Brasil e suas unidades federativas: lista de
espécies. Rev Herpetol Bras.

[B11] Bercovici D, Chudiziniski AM, Dias VDO, Esteves MI, Hiraichi E, Oishi NY (1987). A systematic fractionation of Crotalus durissus terrificus
venom. Mem Inst Butantan.

[B12] Young BA, Kardong KV (2007). Mechanisms Controlling Venom Expulsion in the Western Diamondback
Rattlesnake, Crotalus atrox. J Exp Zool A Ecol Genet Physiol.

[B13] Meier J, Stocker K (1991). Effects of snake venoms on hemostasis. Crit Rev Toxicol.

[B14] Chang CC, Tseng KH (1978). Effect of crotamine, a toxin of South American rattlesnake venom,
on the sodium channel of murine skeletal muscle. Br J Pharmacol.

[B15] Oguiura N, Boni-Mitake M, Rádis-Baptista G (2005). New view on crotamine, a small basic polypeptide myotoxin from
South American rattlesnake venom. Toxicon.

[B16] Gonçalves JM, Polson A (1947). The electrophoretic analysis of snake venoms. Arch Biochem.

[B17] Gonçalves JM, Vieira LG (1950). Estudos sobre venenos de serpentes brasileiras. I. Análise
eletroforético. An Acad Bras Cienc.

[B18] Oguiura N, Camargo ME, Da Silva ARP, Horton DSPQ (2000). Quantification of crotamine, a small basic myotoxin, in South
American rattlesnake (Crotalus durissus terrificus) venom by enzyme-linked
immunosorbent assay with parallel-lines analysis. Toxicon.

[B19] Laure CJ (1975). Die primärstruktur des crotamins. Hoppe-Seyler’s Z Physiol Chem.

[B20] Beltran JR, Mascarenhas YP, Craievich AF, Laure CJ (1990). SAXS study of the snake toxin. Eur Biophys J.

[B21] Dimarcq JL, Bulet P, Hetru C, Hoffmann J (1998). Cysteine-Rich Antimicrobial Peptides in
Invertebrates. Biopolymer.

[B22] Nicastro G, Franzoni L, De Chiara C, Mancin AC, Giglio JR, Spisni A (2003). Solution structure of crotamine, a Na+ channel affecting toxin
from Crotalus durissus terrificus venom. Eur J Biochem.

[B23] Fadel V, Bettendorff P, Herrmann T, De Azevedo WF, Oliveira EB, Yamane T (2005). Automated NMR structure determination and disulfide bond
identification of the myotoxin crotamine from Crotalus durissus
terrificus. Toxicon.

[B24] Cameron DL, Tu AT (1978). Chemical and functional homology of myotoxin a from prairie
rattlesnake venom and crotamine from South American rattlesnake
venom. Biochim Biophys Acta.

[B25] Gonçalves J (1956). Estudos sobre venenos de serpentes brasileiras. II-Crotalus
terrificus crotaminicus, subespécie biológica. An Acad Bras Cien.

[B26] Brazil OV, Fontana MD (1993). Toxins as tools in the study of sodium channel distribution in
the muscle fibre membrane. Toxicon.

[B27] Peigneur S, Orts DJ, Prieto da Silva AR, Oguiura N, Boni-Mitake M, de Oliveira EB (2012). Crotamine pharmacology revisited: novel insights based on the
inhibition of Kv channels. Mol Pharmacol.

[B28] Campeiro JD, Marinovic MP, Carapeto FC, Dal Mas C, Monte GG, Porta LC (2018). Oral treatment with a rattlesnake native polypeptide crotamine
efficiently inhibits the tumor growth with no potential toxicity for the
host animal and with suggestive positive effects on animal metabolic
profile. Amino Acids.

[B29] Hayashi MAF, Nascimento FD, Kerkis A, Oliveira V, Oliveira EB, Pereira A (2008). Cytotoxic effects of crotamine are mediated through lysosomal
membrane permeabilization. Toxicon.

[B30] Nascimento FD, Hayashi MAF, Kerkis A, Oliveira V, Oliveira EB, Rádis-Baptista G (2007). Crotamine mediates gene delivery into cells through the binding
to heparan sulfate proteoglycans. J Biol Chem.

[B31] Costa BA, Sanches L, Gomide AB, Bizerra F, Dal Mas C, Oliveira EB (2014). Interaction of the rattlesnake toxin crotamine with model
membranes. J Phys Chem B.

[B32] da Cunha DB, Silvestrini AVP, da Silva ACG, Estevam DMP, Pollettini FL, Navarro JO (2018). Mechanistic insights into functional characteristics of native
crotamine. Toxicon.

[B33] Passero LF, Tomokane TY, Corbett CE, Laurenti MD, Toyama MH (2007). Comparative studies of the anti-leishmanial activity of three
Crotalus durissus ssp. venoms. Parasitol Res.

[B34] Dal Mas C, Moreira JT, Pinto S, Monte GG, Nering MB, Oliveira EB (2015). Anthelmintic effects of a cationic toxin from a South American
rattlesnake venom. Toxicon.

[B35] Maluf SEC, Dal Mas C, Oliveira EB, Melo PM, Carmona AK, Gazarini ML (2016). Inhibition of malaria parasite Plasmodium falciparum development
by crotamine, a cell penetrating peptide from the snake
venom. Peptides.

[B36] Boldrini-França J, Corrêa-Netto C, Silva MMS, Rodrigues RS, De La Torre P, Pérez A (2010). Snake venomics and antivenomics of Crotalus durissus subspecies
from Brazil: assessment of geographic variation and its implication on
snakebite management. J Proteomics.

[B37] Toyama DO, Boschero AC, Martins MA, Fonteles MC, Monteiro HS, Toyama MH (2005). Structure-function relationship of new crotamine isoform from the
Crotalus durissus cascavella. Protein J.

[B38] Santoro ML, Sousa-e-Silva MCC, Gonçalves LRC, Almeida-Santos SM, Cardoso DF, Laporta-Ferreira IL (1999). Comparison of the biological activities in venoms from three
subspecies of the South American rattlesnake (Crotalus durissus terrificus,
C. durissus cascavella and C. durissus collilineatus). Comp Biochem Physiol C Pharmacol Toxicol Endocrinol.

[B39] Schenberg S (1959). Geographical pattern of crotamine distribution in the same
rattlesnake subspecies. Science.

[B40] Oguiura N, Collares MA, Furtado MFD, Ferrarezzi H, Suzuki H (2009). Intraspecific variation of the crotamine and crotasin genes in
Crotalus durissus rattlesnakes. Gene.

[B41] Calvete JJ, Sanz L, Cid P, de La Torre P, Flores-Díaz M, Dos Santos MC (2010). Snake venomics of the Central American rattlesnake Crotalus simus
and the South American Crotalus durissus complex points to neurotoxicity as
an adaptive paedomorphic trend along Crotalus dispersal in South
America. J Proteome Res.

[B42] Laemmli UK (1970). Cleavage of structural proteins during the assembly of the head
of bacteriophage T4. Nature.

[B43] Harlow E (1988). Immunoblotting. in Antibodies, a Laboratory Manual.

[B44] Calvete JJ, Sanz L, Pérez A, Borges A, Vargas AM, Lomonte B (2011). Snake population venomics and antivenomics of Bothrops atrox:
Paedomorphism along its transamazonian dispersal and implications of
geographic venom variability on snakebite management. J Proteomics.

[B45] LNBio (2008). Protocolo de digestão em solução: Laboratório de Espectrometria de
Massas.

[B46] Distler U, Kuharev J, Navarro P, Levin Y, Schild H, Tenzer S (2014). Drift time-specific collision energies enable deep-coverage
data-independent acquisition proteomics. Nature methods.

[B47] Pedroso AP, Souza AP, Dornellas APS, Oyama LM, Nascimento CMO, Santos GMS (2017). Intrauterine growth restriction programs the hypothalamus of
adult male rats: integrated analysis of proteomic and metabolomic
data. J Proteome Res.

[B48] Abreu TF, Sumitomo BN, Nishiyama MY, Oliveira UC, Souza GHMF, Kitano ES (2017). Peptidomics of Acanthoscurria gomesiana spider venom reveals new
toxins with potential antimicrobial activity. J Proteomics.

[B49] Finney DJ, Tattersfield F (1952). Probit analysis.

[B50] De Oliveira SAM, Magalhães MR, Salazar VCR, Valadares MC, Da Cunha LC (2015). Identification of crotamine in the venom of Crotalus durissus
collilineatus by three different methods. Toxicon.

[B51] Lourenço A, Creste CFZ, de Barros LC, dos Santos LD, Pimenta DC, Barraviera B (2013). Individual venom profiling of Crotalus durissus terrificus
specimens from a geographically limited region: Crotamine assessment and
captivity evaluation on the biological activities. Toxicon.

[B52] Oliveira IS, Cardoso IA, Bordon KCF, Carone SEI, Boldrini-França J, Pucca MB (2018). Global proteomic and functional analysis of Crotalus durissus
collilineatus individual venom variation and its impact on
envenoming. J Proteomics.

[B53] Rangel-Santos A, Dos-Santos EC, Lopes-Ferreira M (2004). A comparative study of biological activities of crotoxin and CB
fraction of venoms from Crotalus durissus terrificus, Crotalus durissus
cascavella and Crotalus durissus collilineatus. Toxicon.

[B54] Freitas-de-Sousa LA, Amazonas DR, Sousa LF, Sant’Anna SS, Nishiyama MY, Serrano SMT (2015). Comparison of venoms from wild and long-term captive Bothrops
atrox snakes and characterization of Batroxrhagin, the predominant class
PIII metalloproteinase from the venom of this species. Biochimie.

[B55] McCleary RJR, Sridharan S, Dunstan NL, Mirtschin PJ, Kini RM (2016). Proteomic comparisons of venoms of long-term captive and recently
wild-caught Eastern brown snakes (Pseudonaja textilis) indicate venom does
not change due to captivity. J Proteomics.

[B56] de Farias IB, de Morais-Zani K, Serino-Silva C, Sant’Anna SS, da Rocha MMT, Grego KF (2018). Functional and proteomic comparison of Bothrops jararaca venom
from captive specimens and the Brazilian Bothropic Reference
Venom. J Proteomics.

[B57] Galizio NC, Serino-Silva C, Stuginski DR, Abreu PAE, Sant’Anna SS, Grego KF (2018). Compositional and functional investigation of individual and
pooled venoms from long-term captive and recently wild-caught Bothrops
jararaca snakes. J Proteomics.

[B58] Teixeira-araújo R, Castanheira P, Brazil-Más L, Pontes F, De Araújo ML, Lucia M (2017). Antivenomics as a tool to improve the neutralizing capacity of
the crotalic antivenom: a study with crotamine. J Venom Anim Toxins incl Trop Dis.

[B59] Corrêa-Netto C, Junqueira-de-Azevedo IDLM, Silva DA, Ho PL, Leitão-de-Araújo M, Lúcia M (2011). Snake venomics and venom gland transcriptomic analysis of
Brazilian coral snakes, Micrurus altirostris and M.
corallines. J Proteomics.

[B60] Lomonte B, Sasa M, Rey-Suárez P, Bryan W, Gutiérrez JM (2016). Venom of the coral snake Micrurus clarki: proteomic profile,
toxicity, immunological cross-neutralization, and characterization of a
three-finger toxin. Toxins (Basel).

[B61] Queiroz GP, Pessoa LA, Portaro FCV, Furtado MFD, Tambourgi DV (2008). Interspecific variation in venom composition and toxicity of
Brazilian snakes from Bothrops genus. Toxicon.

[B62] Tanaka GD, Sant’Anna A, Marcelino JR, da Luz ACL, da Rocha MMT, Tambourgi DV (2016). Micrurus snake species: venom immunogenicity, antiserum
cross-reactivity and neutralization potential. Toxicon.

[B63] Xiong S, Huang C (2018). Synergistic strategies of predominant toxins in snake
venoms. Toxicol Lett.

[B64] Guo C, Liu S, Yao Y, Zhang Q, Sun MZ (2012). Past decade study of snake venom L-amino acid
oxidase. Toxicon.

[B65] Costa TR, Burin SM, Menaldo DL, de Castro FA, Sampaio SV (2014). Snake venom L-amino acid oxidases: an overview on their antitumor
effects. J Venom Anim Toxins incl Trop Dis.

[B66] Wiezel GA, Rustiguel JK, Morgenstern D, Zoccal KF, Faccioli LH, Nonato MC (2019). Insights into the structure, function and stability of
bordonein-L, the first L-amino acid oxidase from Crotalus durissus
terrificus snake venom. Biochimie.

[B67] Nogueira MF, Barrella TH, Silva RD, Lopes CAM, Araújo JP (2002). Isolation of an Ophidian Paramyxovirus (OPMV) in a captive
rattlesnake (Crotalus durissus terrificus) from Botucatu, São Paulo State,
Brazil. J Venom Anim Toxins.

[B68] Mancin AC, Soares AM, Andrião-Escarso SH, Faça VM, Greene LJ, Zuccolotto S (1998). The analgesic activity of crotamine, a neurotoxin from Crotalus
durissus terrificus (South American rattlesnake) venom: a biochemical and
pharmacological study. Toxicon.

[B69] Boni-Mitake M, Costa H, Spencer PJ, Vassilieff VS, Rogero JR (2001). Effects of 60Co gamma radiation on crotamine. Braz J Med Biol Res.

[B70] Oshima-Franco Y, Hyslop S, Prado-Franceschi J, Cruz-Höfling MA, Rodrigues-Simioni L (1999). Neutralizing capacity of antisera raised in horses and rabbits
against Crotalus durissus terrificus (South American rattlesnake) venom and
its main toxin, crotoxin. Toxicon.

[B71] Ministério da Saúde (1996). Normas técnicas de produção e controle de qualidade, Portaria
174.

