# Supplementary material for: Crotamine in Crotalus durissus: distribution according to subspecies and geographic origin, in captivity or nature
Source: J Venom Anim Toxins Incl Trop Dis. 2020 Apr 6;26:e20190053. doi: 10.1590/1678-9199-JVATITD-2019-0053 (PMC7187639; doi:10.1590/1678-9199-JVATITD-2019-0053)
Supplement: Additional file 2. [file 1678-9199-jvatitd-26-e20190053-s2.pdf]

# Supplementary material to "Crotamine in *Crotalus durissus*: distribution according to subspecies and geographic origin, in captivity or nature"

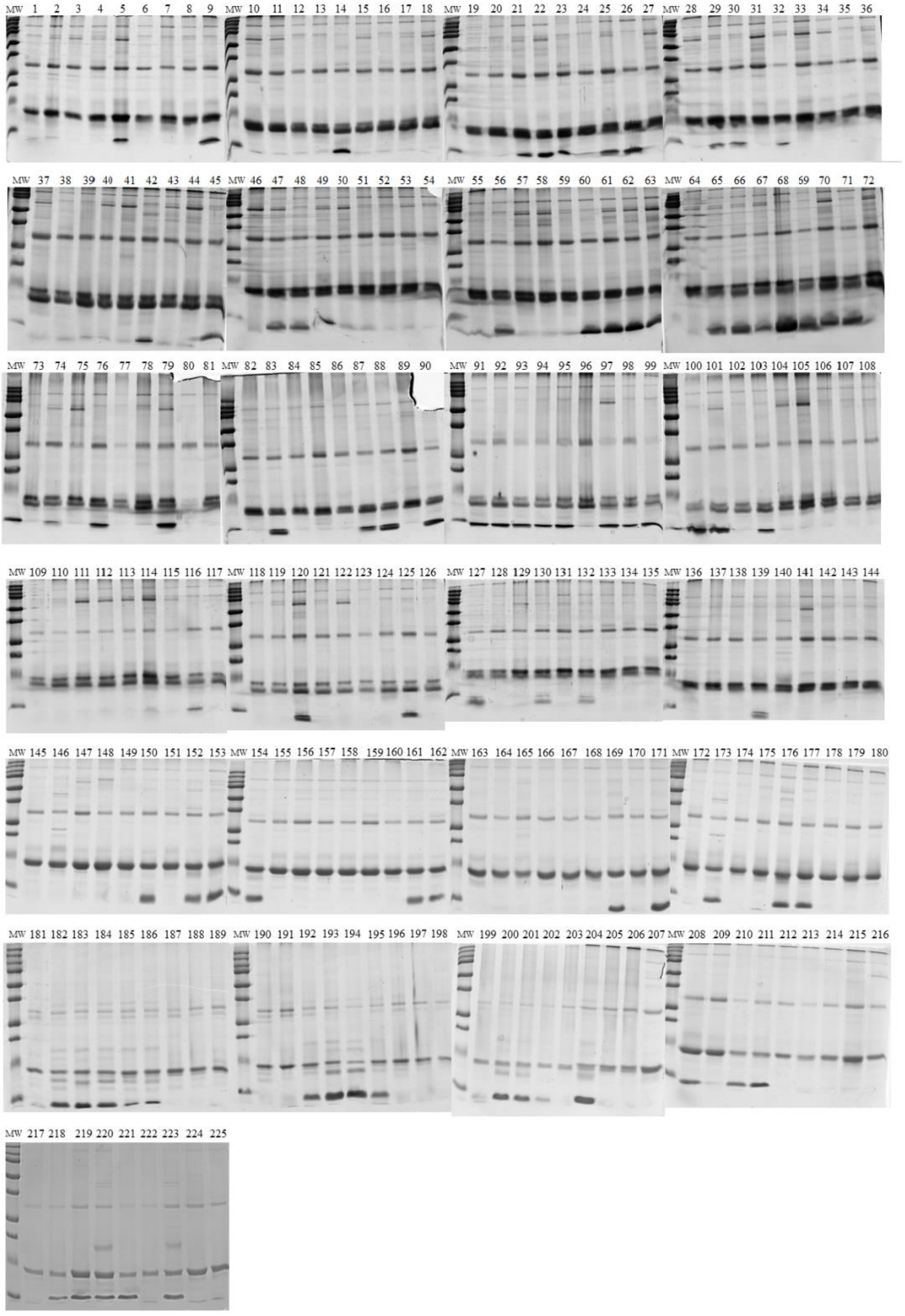


**Additional file 2.** Polyacrylamide gel electrophoresis (15%) of all *C. durissus* used in this work. The numbers of samples correspond to snakes informed in Additional file 1.
